# Design, Synthesis, and Biological Evaluation of 2-(Benzylamino-2-Hydroxyalkyl)Isoindoline-1,3-Diones Derivatives as Potential Disease-Modifying Multifunctional Anti-Alzheimer Agents

**DOI:** 10.3390/molecules23020347

**Published:** 2018-02-07

**Authors:** Dawid Panek, Anna Więckowska, Anna Pasieka, Justyna Godyń, Jakub Jończyk, Marek Bajda, Damijan Knez, Stanislav Gobec, Barbara Malawska

**Affiliations:** 1 Department of Physicochemical Drug Analysis, Faculty of Pharmacy, Jagiellonian University Medical College, Medyczna 9, 30-688 Kraków, Poland; dawid.panek@doctoral.uj.edu.pl (D.P.); anna.wieckowska@uj.edu.pl (A.W.); pasieka.ann@gmail.com (A.P.); justyna.godyn@doctoral.uj.edu.pl (J.G.); jakub.jonczyk@doctoral.uj.edu.pl (J.J.); marek.bajda@uj.edu.pl (M.B.); 2 Department of Pharmaceutical Chemistry, Faculty of Pharmacy, University of Ljubljana, Aškerčeva 7, 1000 Ljubljana, Slovenia; damijan.knez@ffa.uni-lj.si (D.K.); stanislav.gobec@ffa.uni-lj.si (S.G.)

**Keywords:** isoindoline-1,3-dione derivatives, cholinesterase inhibitors, BACE-1 inhibitors, Aβ-aggregation, molecular modeling, multiple anti-Alzheimer’s ligands

## Abstract

The complex nature of Alzheimer’s disease calls for multidirectional treatment. Consequently, the search for multi-target-directed ligands may lead to potential drug candidates. The aim of the present study is to seek multifunctional compounds with expected activity against disease-modifying and symptomatic targets. A series of 15 drug-like various substituted derivatives of 2-(benzylamino-2-hydroxyalkyl)isoindoline-1,3-diones was designed by modification of cholinesterase inhibitors toward β-secretase inhibition. All target compounds have been synthesized and tested against eel acetylcholinesterase (*ee*AChE), equine serum butyrylcholinesterase (*eq*BuChE), human β-secretase (*h*BACE-1), and β-amyloid (Aβ-aggregation). The most promising compound, **12** (2-(5-(benzylamino)-4-hydroxypentyl)isoindoline-1,3-dione), displayed inhibitory potency against *ee*AChE (IC_50_ = 3.33 μM), *h*BACE-1 (43.7% at 50 μM), and Aβ-aggregation (24.9% at 10 μM). Molecular modeling studies have revealed possible interaction of compound **12** with the active sites of both enzymes—acetylcholinesterase and β-secretase. In conclusion: modifications of acetylcholinesterase inhibitors led to the discovery of a multipotent anti-Alzheimer’s agent, with moderate and balanced potency, capable of inhibiting acetylcholinesterase, a symptomatic target, and disease-modifying targets: β-secretase and Aβ-aggregation.

## 1. Introduction

Alzheimer’s disease (AD) is one of the neurodegenerative diseases and the most commonly diagnosed cause of dementia in the elderly. It affects an estimated 36 million people worldwide, and this figure is expected to increase to 100 million by the end of 2050 [1]. The lack of an effective disease-modifying treatment for AD, caused, among others, by the disease’s poorly understood and complex etiopathogenesis, is a major challenge facing modern medicine. Deposits of insoluble proteins: β-amyloid (Aβ) and hyperphosporylated tau are regarded as the primary cause of AD. Aβ is the product of enzymatic cleavage of amyloid precursor protein (APP) by β-secretase (BACE-1) and γ-secretase. Various forms of Aβ, primarily Aβ_1–42_ and Aβ_1–40_, have the ability to aggregate and create extracellular neurotoxic senile plaques [2]. In parallel, inside neurons, excessive phosphorylation of the tau protein leads to the formation of neurofibrillary tangles [3]. Both processes may begin up to 25 years before initial symptoms occur [4]. As complementary elements of the etiopathogenesis of AD, they represent obvious biological targets in search for anti-AD treatments. Progressive neurodegeneration in the course of AD affects mainly cholinergic neurotransmission [5,6,7]. Current pharmacotherapy of AD is therefore based on the restoration of cholinergic activity with cholinesterase inhibitors: donepezil, rivastigmine, and galantamine. These drugs do not prevent, reverse, or even slow down the neurodegenerative processes, but offer only symptomatic treatment based on improving cognition and memory [8]. Therefore, the overriding objective for researchers is to find new, effective disease-modifying agents for the treatment of AD. Complex diseases, such as AD, require multidirectional treatment. An interesting form of polypharmacology involves multi-target-directed-ligands (MTDLs), which are compounds that act on two or more biological targets and mechanisms [9]. This approach has developed intensively over the last few years, particularly in the context of multifactorial diseases such as AD [10,11,12,13]. A large number of potential multifunctional anti-AD molecules has been designed and synthesized by combining chemical fragments responsible for interaction with desirable biological targets [14,15,16,17,18]. In this paper, we present the design, synthesis, biological evaluation, and molecular modeling studies of new phthalimide derivatives linked by a hydroxyalkyl chain with a benzylamine moiety, as novel, potential, multifunctional anti-AD agents. Biological profiling of these new compounds includes the assessment of acetylcholinesterase from *Electrophorus electricus* (*ee*AChE), butyrylcholinesterase from equine serum (*eq*BuChE), BACE-1, and Aβ aggregation inhibition.

## 2. Results and Discussion

### 2.1. Design

**Figure 1:**
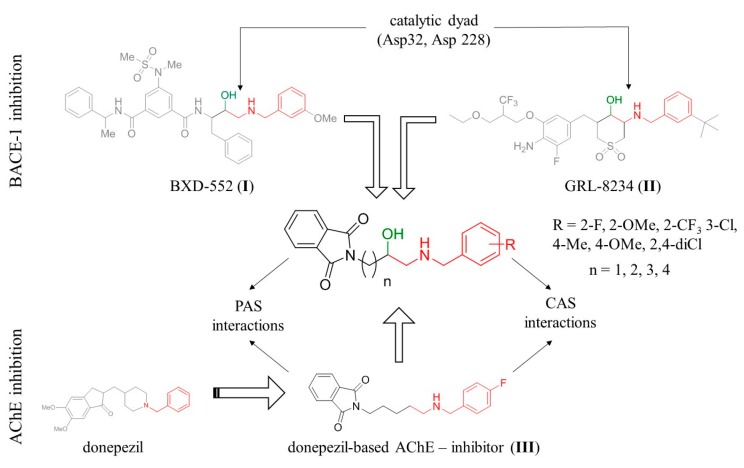
Design strategy for multitarget anti-Alzheimer’s agents. CAS: catalytic anionic site, PAS: peripheral anionic site.

Our main area of interest is the development of new, multifunctional compounds which target the causes of AD. Previously, we have reported a series of dual binding site donepezil-based inhibitors of AChE with anti-Aβ aggregating properties. Compound **III** (Figure 1) was found to be a potent and selective human AChE inhibitor (IC_50_ = 0.268 μM) with anti-Aβ aggregation activity (66% at 10 μM) and a neuroprotective effect against Aβ toxicity at 1 μM and 3 μM [19]. Molecular modeling studies revealed that the phthalimide fragment of compound **III** is responsible for interactions with the peripheral anionic site (PAS), while the benzylamine moiety interacts with the catalytic anionic site of AChE. The goal of the presented study was modification of lead **III** to introduce additional inhibitory activity toward BACE-1. A 2-(benzylamino)ethan-1-ol fragment is frequently incorporated in potent BACE-1 inhibitors; among others, NVP-BXD552 (**I**) [20] and GRL-8234 (**II**) [21] (Figure 1). Therefore, we introduced a hydroxyl group in the alkyl chain of compound **III** and assumed that the resulting hydroxyethylamine fragment will mediate interaction with the catalytic dyad of BACE-1 (composed of two aspartic residues: Asp32 and Asp228), which is the key to the effective inhibition of BACE-1. Moreover, we calculated the most important physicochemical parameters for the designed compounds and confirmed that they meet the criteria set by Lipinski’s rule of five [22] and possess drug-like properties.

### 2.2. Chemistry

**Scheme 1:**
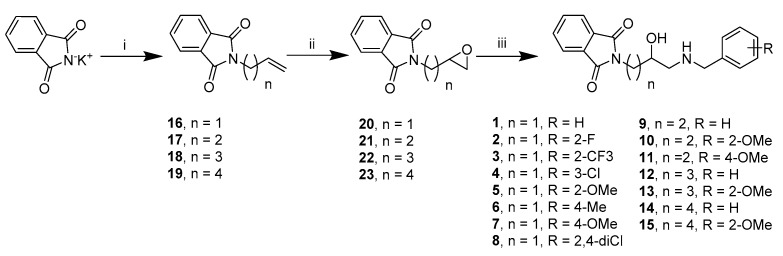
Synthesis of compounds **1**–**15**. Reagents and conditions: (**i**) alkenyl halide, DMF, 80 °C, 16 h; (**ii**) *meta*-chloroperoxybenzoic acid, DCM, room temperature (R.T.) 16 h; (**iii**) benzylamine derivatives, *n*-propanol, pyridine, 97 °C.

We synthesized 15 derivatives with various substituents in the benzylamine fragment and different linker lengths based on results of molecular modeling. The general procedure for the synthesis of the final compounds (**1**–**15**) is presented in Scheme 1. Alkylation of potassium phthalimide by ω-bromobut-1-ene derivatives and subsequent oxidation with *meta*-chloroperoxybenzoic acid (*m*CBPA) yielded epoxides (**20**–**23**). These epoxides were then used as alkylating agents in a reaction with the appropriate benzylamines, leading to final compounds (**1**–**15**) in their racemic form.

### 2.3. Biological Evaluation

Target compounds **1**–**15** were subjected to biological screening to test their inhibitory activity against acetyl- and butyrylcholinesterase, β-secretase, and aggregation of Aβ. We evaluated the potency of the compounds against cholinesterases in Ellman’s assay [23] using AChE from *Electrophorus electricus* (*ee*AChE) and BuChE from equine serum (*eq*BuChE). We used tacrine and donepezil as reference compounds. Following initial screening at 10 μM, we determined IC_50_ values for compounds which exhibited potency greater than 50%. Eleven out of the fifteen compounds display percentages of inhibition of *ee*AChE between <10% and 33% at a concentration of 10 μM (Table 1). Similarly, fourteen out of the fifteen compounds display % inhibition of *eq*BuChE between <10% and 36% at a concentration of 10 μM. Four compounds exhibit moderate *ee*AChE activity (low micromolar IC_50_) and one compound exhibits moderate *eq*BuChE inhibitory (low micromolar IC_50_). The most potent AChE inhibitors, with IC_50_ ranging from 1.95 to 11.07 μM, were compounds **12**–**15** with longer 3- and 4-carbon atom linkers. This observation is similar to our previous findings [19] concerning phthalimide-benzylamine derivatives as cholinesterase inhibitors (compound **III**). The optimal linker length, ensuring interaction with both catalytic anionic site CAS and PAS, was five carbon atoms (compound **III**), corresponding to *n* = 3 in compounds discussed in this work. Compounds with shorter linkers (*n* = 1 and *n* = 2) are weak inhibitors of *ee*AChE (11.5–32.7% inhibition at 10 μM), or are altogether inactive (<10% inhibition). We have not observed any structure-activity relationship regarding substituents at the benzylamine ring; however, we only synthesized differently substituted derivatives with the shortest linkers. Regarding inhibition of *eq*BuChE, we found compound **5**, for which we determined the IC_50_ value (7.86 μM). Unlike the case of AChE inhibition, the most potent inhibitor of BuChE was those compounds with shorter carbon linkers (*n* = 1). Comparing the unsubstituted benzylamine derivative with the 2-methoxy substituted one (**1** versus **5**) we conclude that this substitution is important for BuChE inhibitory activity. The results obtained show that introduction of the hydroxyl group to the alkyl chain led to a reduction in inhibitory activity with respect to both cholinesterase compared to the model compound **III**.

To determine the inhibitory activity of the reported compounds against human recombinant BACE-1 (*h*BACE-1), we used a biochemical spectrofluorometric assay (fluorescence resonance energy transfer, FRET-based) [24,25]. The assay is based on the cleavage of an amyloid precursor protein APP analogue with the Swedish mutation, leading to an increase in fluorescence. As a reference, we included inhibitor **IV** and established its IC_50_ value at 46 nM, which is in accordance with published data [26]. We tested each compound at 50 μM concentrations and calculated their inhibitory activity, which ranged from 15.7% for **15** to 45.0% for **6** (Table 1).

**Figure 2:**
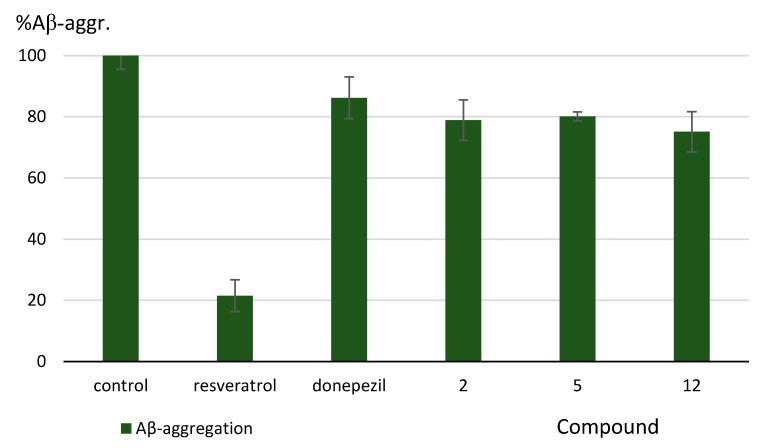
Effect of the synthesized compounds on Aβ aggregation. Values are expressed as means ± standard deviation (SD) for at least two independent experiments.

We determined Aβ antiaggregating properties for the presented compounds (**1**–**15**) in a thioflavin-T assay [27]. The assay was performed at 10 μM inhibitor concentration. Donepezil was used as the reference, since the presented compounds had been structurally developed from this drug. We also included resveratrol as a much stronger inhibitor of Aβ aggregation (Table 1). We found that three of the tested compounds significantly inhibit Aβ aggregation: **2**, **5**, and **12**, with inhibition percentages of 21.1%, 19.9%, and 24.9%, respectively (at 10 μM) (Figure 2). These results are inferior to resveratrol but superior to donepezil.

### 2.4. Molecular Modeling Studies

**Figure 3:**
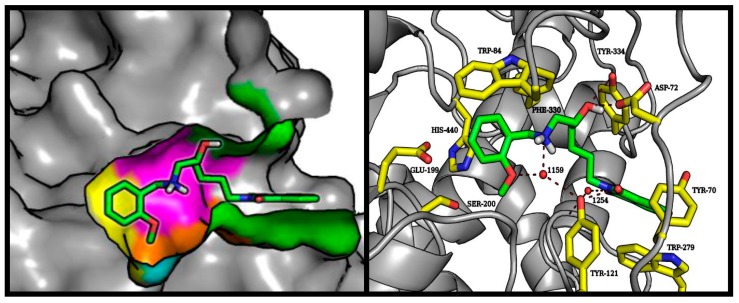
Left panel: illustrative location of compound **15** (green sticks) in the active site of AChE. Active site elements are color-coded: yellow: catalytic triad; magenta: anionic site; orange: acyl pocket; cyan: oxyanion hole; green: PAS. Right panel: detailed visualization of compound **15** (green) interactions with amino acids (yellow) belonging to the active site of AChE, including the conserved waters (red balls).

The presented active compounds were docked into the *Torpedo californica* AChE crystal structure, as well as into human BuChE and human BACE-1. Docking was performed in order to determine the causes of potency variations, by finding differences in the potential bonding mode. We used previously developed methods to dock ligands and assess the binding modes [28,29]. In the case of AChE, the length of the linker had a significant influence on ligand arrangement in the enzymatic active gorge and on docking score value (from 34.01 for inactive compound **3** to 44.47 for active compound **14**. The presence of a hydroxyl group within the linker made it very difficult for the compounds to adjust to the AChE active site. Short linkers (*n* = 1 and *n* = 2) were halted within the PAS by hydrogen bonds generated by OH with Tyr334 and Asp72, restricting interactions between benzylamine and CAS or between phthalimide and PAS. As the linker grows in length, the effect of the hydroxyl group is compensated for by the flexibility of the compound. The binding mode of the most active inhibitor **15** is shown in Figure 3.

Despite hydrogen bonding of the hydroxyl group with Tyr334 and Asp72 at the proximal part of the active gorge, this compound adopts a conformation which resembles potent donepezil-like AChE inhibitors. The first key element, is the benzylamine position, providing CH-π interaction with Trp84 and cation-π interactions with Phe330. Hydrogen bonds between the ligand and the conserved water molecule (1159) appear to be significant. The most active compound, with the longest carbon linker, also provides the best phthalimide-PAS fit. This was the only compound which formed both hydrogen bonds, with Tyr121 and conserved water molecule (1254), while maintaining optimal π-π interaction with Trp279 and CH-π interaction with Tyr-70.

**Figure 4:**
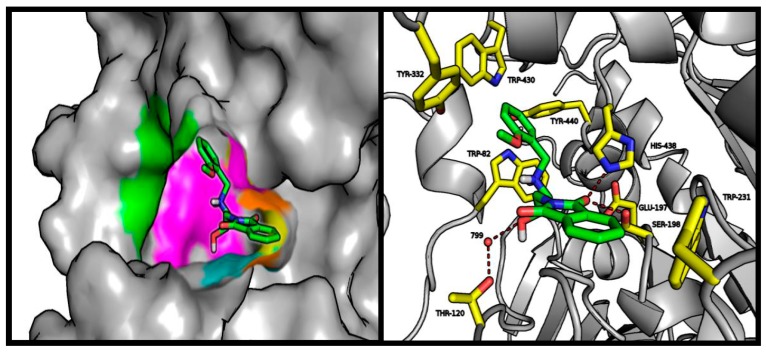
Left panel: illustrative location of compound **5** (green sticks) in the active site of BuChE. Active site elements are color-coded: yellow: catalytic triad; magenta: anionic site; orange: acyl pocket; cyan: oxyanion hole; green: PAS. Right panel: detailed visualization of compound **5** (green) interactions with amino acids (yellow) belonging to the active site of AChE, including the conserved water (red balls).

The predicted BuChE binding mode for active compound (**5**) was very consistent despite differences observed in biological studies. Interactions with three tryptophan residues—Trp82, Trp231, and Trp430—appeared to be crucial from the point of view of the molecular modeling results. Similarly to BuChE substrates, the tested compound exhibited cation-π interactions between the protonated amine basic center and Trp82 [30]. Phthalimide, in a manner analogous to the BuChE-decomposed ester, occupied a position close to CAS. The active compound (**5**) provides a good illustration of the presented binding mode (Figure 4). The carbonyl oxygen atom of phthalimide is involved in the hydrogen bonding network of Ser198 and His438. Depending on the analyzed enantiomer, the short linker may facilitate binding of the hydroxyl group with the conserved HOH799 water molecule, and through it, with Thr120 (*S*-enantiomer shown in Figure 4). The R-enantiomer hydroxyl group interacts with Glu197. Cation-π interactions of the protonated amine are stabilized by hydrophobic and aromatic interactions of the aromatic benzyl ring with the hydrophobic pocket formed by Tyr332, Trp430, and Tyr440. Interactions of the methoxy group with Tyr332 are clearly preferred.

**Figure 5:**
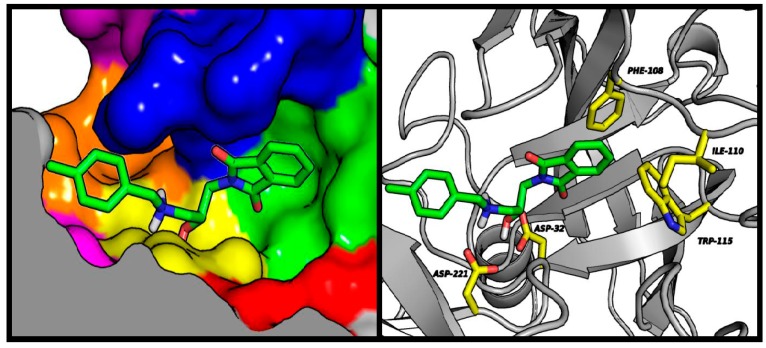
Left panel: illustrative location of compound **6** (green sticks) in the active site of BACE-1. Active site elements are color-coded: S3′: violet; S2′: orange; S1′: magenta; S1: yellow; S2: blue; S3: green; S4: red. Right panel: detailed visualization of compound 6 (green sticks) interactions with amino acids (yellow) belonging to the active site of BACE-1.

Despite the addition of a hydroxyethylamine moiety associated with the benzyl substituent, which is a characteristic pharmacophore in BACE-1 inhibitors, the tested compounds did not exhibit strong enzymatic inhibition potency. Molecular modeling studies indicate that the described pharmacophore binds at the preferred place (catalytic dyad consisting of Asp32 and Asp221); however, there is a very broad variation in the predicted conformation of both aromatic systems. The good fit of the phthalimide fragment within the S3 hydrophobic pocket makes it difficult to direct the benzylic substituent towards the S2′ pockets. This situation is illustrated in Figure 5. Compound **6** showed the highest-rated conformation in the BACE-1 active site. In other conformations obtained during docking experiments, the benzylamine and hydroxyethyl moieties assumed the same position as the corresponding reference compound (NVP-BXD552); however, under these conditions phthalimide provides only weak interaction beyond the S3 sub-pocket.

## 3. Materials and Methods

### 3.1. Chemistry 

#### 3.1.1. General Methods

^1^H NMR and ^13^C NMR spectra were recorded on a Varian Mercury 300 (Varian, San Diego, CA, USA) at 300 MHz. The chemical shifts for ^1^H NMR are referenced to tetramethylsilane TMS via residual solvent signals (^1^H, CDCl_3_ at 7.26 ppm, DMSO-*d*_6_ at 2.50 ppm). Mass spectra (MS) were recorded on a UPLC-MS/MS system consisting of a Waters ACQUITY^®^ UPLC^®^ (Waters Corporation, Milford, MA, USA) coupled to a Waters TQD mass spectrometer (electrospray ionization mode ESI-tandem quadrupole). Analytical thin layer chromatography (TLC) was done using aluminum sheets precoated with silica gel 60 F_254_. Column chromatography was performed on Merck silica gel 60 (63–200 μm) (Merck, Darmstadt, Germany). For the TLC and column chromatography, the following solvents were used: dichloromethane (DCM), methanol (MeOH), petroleum ether, chloroform, ethyl acetate, and 25% ammonia water solution. The purity of the final compounds was determined using an analytical RPLC-MS on a Waters Acquity TQD using an Aquity UPLC BEH C18 column (1.7 μm, 2.1 × 100 mm) at 214 nm and 254 nm. CH_3_CN/H_2_O gradient with 0.1% HCOOH was used as the mobile phase at a flow rate of 0.3 mL/min. All of the compounds showed purity above 95%. All of the reagents were purchased from commercial suppliers and were used without further purification. The following compounds: 2-allylisoindoline-1,3-dione (**16**) [31], 2-(but-3-en-1-yl)isoindoline-1,3-dione (**17**) [32], 2-(pent-4-en-1-yl)isoindoline-1,3-dione (**18**) [33], 2-(hex-5-en-1-yl)isoindoline-1,3-dione (**19**) [34], 2-(oxiran-2-ylmethyl)isoindoline-1,3-dione (**20**) [35], 2-(2-(oxiran-2-yl)ethyl)isoindoline-1,3-dione (**21**) [35], 2-(3-(oxiran-2-yl)propyl)isoindoline-1,3-dione (**22**) [35], 2-(4-(oxiran-2-yl)butyl)isoindoline-1,3-dione (**23**) [36] have been reported previously.

#### 3.1.2. General Procedure for the Synthesis of Compounds: **1**–**15** (Procedure A)

2-(ω-(oxiranyl)alkyl)isoindoline-1,3-dione (**21**) (1.0 equiv.), corresponding benzylamine (1.0 equiv.), and a catalytic amount of pyridine in *n*-propanol were refluxed for 16 h. Then, the solvent was evaporated and the resulting residue was purified by flash column chromatography using a mixture of MeOH and DCM (gradient from 1% to 10% of MeOH).

*2-(3-(Benzylamino)-2-hydroxypropyl)isoindoline-1,3-dione* (**1**). Following the procedure A, reaction of phenylmethanamine (0.065 mL, 0.591 mmoL) with 2-(oxiran-2-ylmethyl)isoindoline-1,3-dione (**20**) (0.120 g, 0.591 mmoL) and a catalytic amount of pyridine in 4 mL *n*-propanol was performed. Yield: 36 mg (19.6%), TLC (DCM/MeOH, 9.5/0.5, *v*/*v*) R_f_ = 0.33. MW 310.35. Formula C_18_H_18_N_2_O_3_. MS *m*/*z* 311.09 (M + H^+^). ^1^H NMR (300 MHz, CDCl_3_) δ 7.80–7.89 (m, 2H), 7.66–7.76 (m, 2H), 7.18–7.38 (m, 5H), 3.98 (tdd, *J* = 6.92, 5.26, 3.98 Hz, 1H), 3.69–3.87 (m, 4H), 2.79 (dd, *J* = 12.31, 3.85 Hz, 1H), 2.65 (dd, *J* = 12.31, 7.18 Hz, 1H), 2.33 (br.s., 2H). ^13^C NMR (75 MHz, CDCl_3_) δ 168.67, 139.62, 134.05, 131.97, 128.47, 128.14, 127.16, 123.38, 68.05, 53.75, 51.84, 41.91.

*2-(3-((2-Fluorobenzyl)amino)-2-hydroxypropyl)isoindoline-1,3-dione* (**2**). Following the procedure A, reaction of (2-fluorophenyl)methanamine (0.068 mL, 0.591 mmoL) with 2-(oxiran-2-ylmethyl)isoindoline-1,3-dione (**20**) (0.120 g, 0.591 mmoL) and a catalytic amount of pyridine in 4 mL *n*-propanol was performed. Yield: 50 mg (26.0%), TLC (DCM/MeOH, 9.5/0.5, *v*/*v*) R_f_ = 0.21. MW 328.34. Formula C_18_H_17_FN_2_O_3_. MS *m*/*z* 329.10 (M + H^+^). ^1^H NMR (300 MHz, CDCl_3_) δ 7.79–7.88 (m, 2H), 7.66–7.75 (m, 2H), 7.35 (td, *J* = 7.50, 1.67 Hz, 1H), 7.19–7.29 (m, 1H), 7.06–7.14 (m, 1H), 7.02 (ddd, *J* = 10.00, 8.46, 1.03 Hz, 1H), 3.98–4.10 (m, 1H), 3.90 (s, 2H), 3.81 (dd, *J* = 14.11, 6.92 Hz, 1H), 3.73 (dd, *J* = 13.85, 4.87 Hz, 1H), 3.49 (br.s., 2H), 2.81 (dd, *J* = 12.31, 3.85 Hz, 1H), 2.66 (dd, *J* = 12.05, 7.44 Hz, 1H). ^13^C NMR (75 MHz, CDCl_3_) δ 168.03, 162.21, 133.44, 131.35, 129.99 (d, *J_C=F_* = 1.9 Hz), 128.60 (d, *J_C=F_* = 8.29 Hz), 125.04 (d, *J_C=F_* = 14.93 Hz), 123.61 (d, *J_C=F_* = 3.32 Hz), 122.78, 114.78 (d, *J_C=F_* = 22.12 Hz), 67.11, 50.97, 46.23, 41.23.

*2-(2-Hydroxy-3-((2-(trifluoromethyl)benzyl)amino)propyl)isoindoline-1,3-dione* (**3**). Following the procedure A, reaction of (2-(trifluoromethyl)phenyl)methanamine (0.270 mL, 1.969 mmoL) with 2-(oxiran-2-ylmethyl)isoindoline-1,3-dione (**20**) (0.400 g, 1.969 mmoL) and a catalytic amount of pyridine in 5 mL *n*-propanol was performed. Yield: 150 mg (20.1%), TLC (DCM/MeOH, 9.5/0.5, *v*/*v*) R_f_ = 0.16. MW 378.35. Formula C_19_H_17_F_3_N_2_O_3_. MS *m*/*z* 379.08 (M + H^+^). ^1^H NMR (300 MHz, CDCl_3_) δ 7.81–7.90 (m, 2H), 7.69–7.77 (m, 2H), 7.62 (t, *J* = 8.50 Hz, 2H), 7.52 (t, *J* = 7.40 Hz, 1 H), 7.35 (t, *J* = 7.20 Hz, 1H), 3.94–4.05 (m, 3H), 3.85 (dd, *J* = 14.11, 6.67 Hz, 1H), 3.77 (dd, *J* = 13.85, 5.13 Hz, 1H), 2.83 (dd, *J* = 12.05, 4.10 Hz, 1H), 2.68 (dd, *J* = 12.05, 7.18 Hz, 1H), 2.23 (br.s., 2H) ^13^C NMR (75 MHz, CDCl_3_) δ 168.70, 138.43 (d, *J_C=F_* = 1.11 Hz), 134.08, 132.11, 130.31, 128.50, 127.08, 126.28, 125.94 (*J_C=F_* = 6.08 Hz), 123.40, 122.64, 68.25, 52.14, 49.84 (d, *J_C=F_* = 2.21 Hz), 41.89.

*2-(3-((3-Chlorobenzyl)amino)-2-hydroxypropyl)isoindoline-1,3-dione* (**4**). Following the procedure A, reaction of (3-chlorophenyl)methanamine (0.090 mL, 0.738 mmoL) with 2-(oxiran-2-ylmethyl)isoindoline-1,3-dione (**20**) (0.150 g, 0.738 mmoL) and a catalytic amount of pyridine in 4.5 mL *n*-propanol was performed. Yield: 75 mg (29.5%), TLC (DCM/MeOH, 9.5/0.5, *v*/*v*) R_f_ = 0.22. MW 344.80. Formula C_18_H_17_ClN_2_O_3_. MS *m*/*z* 345.62 (M + H^+^). ^1^H NMR (300 MHz, CDCl_3_) δ 7.81–7.89 (m, 2H), 7.69–7.77 (m, 2H), 7.28–7.33 (m, 1H), 7.14–7.28 (m, 3H), 3.93–4.05 (m, 1H), 3.71–3.89 (m, 4H), 2.77 (dd, *J* = 12.31, 3.85 Hz, 1H), 2.64 (dd, *J* = 12.31, 6.92 Hz, 1H), 2.31 (br.s., 2H). ^13^C NMR (75 MHz, CDCl_3_) δ 168.72, 141.99, 134.28, 134.10, 131.92, 129.69, 128.16, 127.24, 126.17, 123.41, 68.30, 53.26, 51.88, 41.91.

*2-(2-Hydroxy-3-((2-methoxybenzyl)amino)propyl)isoindoline-1,3-dione* (**5**). Following the procedure A, reaction of (2-methoxyphenyl)methanamine (0.096 mL, 0.738 mmoL) with 2-(oxiran-2-ylmethyl)isoindoline-1,3-dione (**20**) (0.150 g, 0.738 mmoL) and a catalytic amount of pyridine in 4.5 mL *n*-propanol was performed. Yield: 66 mg (26.3%), TLC (DCM/MeOH, 9.5/0.5, *v*/*v*) R_f_ = 0.29. MW 340.38. Formula C_19_H_20_N_2_O_4_. MS *m*/*z* 341.12 (M + H^+^). ^1^H NMR (300 MHz, CDCl_3_) δ 7.83–7.99 (m, 2H), 7.57–7.71 (m, 2H), 7.15–7.23 (m, 1H), 7.09 (dd, *J* = 7.57, 1.41 Hz, 1H), 6.80–6.89 (m, 2H), 3.80–4.11 (m, 7H), 3.63–3.72 (m, 1H), 3.57 (dd, *J* = 11.03, 4.62 Hz, 1H), 2.62–3.01 (m, 2H). ^13^C NMR (75 MHz, CDCl_3_) δ 167.52, 156.76, 132.98, 132.80, 131.94, 129.72, 128.32, 128.19, 125.44, 123.79, 120.54, 110.58, 70.78, 64.58, 55.61, 52.12, 36.79.

*2-(2-Hydroxy-3-((4-methylbenzyl)amino)propyl)isoindoline-1,3-dione* (**6**). Following the procedure A, reaction of (4-(methyl)phenyl)methanamine (0.063 mL, 121.18 mmoL) with 2-(oxiran-2-ylmethyl)isoindoline-1,3-dione (**20**) (0.100 g, 0.492 mmoL) and a catalytic amount of pyridine in 3.5 mL *n*-propanol was performed. Yield: 47 mg (29.5%), TLC (DCM/MeOH, 9.5/0.5, *v*/*v*) R_f_ = 0.22. MW 324.38. Formula C_19_H_20_N_2_O_3_. MS *m*/*z* 325.11 (M + H^+^). ^1^H NMR (300 MHz, CDCl_3_) δ 7.78–7.89 (m, 2H), 7.68–7.72 (m, 2H), 7.06–7.21 (m, 4H), 3.93–4.03 (m, 1H), 3.63–3.86 (m, 4H), 3.00 (br.s., 2H), 2.76 (dd, *J* = 12.31, 3.85 Hz, 1H), 2.62 (dd, *J* = 12.31, 7.44 Hz, 1H), 2.30 (s, 3H). ^13^C NMR (75 MHz, CDCl_3_) δ 168.64, 136.75, 136.48, 134.01, 131.97, 129.15, 128.14, 123.35, 67.93, 53.41, 51.79, 41.92, 21.07.

*2-(2-Hydroxy-3-((4-methoxybenzyl)amino)propyl)isoindoline-1,3-dione* (**7**). Following the procedure A, reaction of (4-methoxyphenyl)methanamine (0.096 mL, 0.738 mmoL) with 2-(oxiran-2-ylmethyl)isoindoline-1,3-dione (**20**) (0.150 g, 0.738 mmol) and a catalytic amount of pyridine in 4.5 mL *n*-propanol was performed. Yield: 92 mg (36.6%), TLC (DCM/MeOH, 9.5/0.5, *v*/*v*) R_f_ = 0.27. MW 340.38. Formula C_19_H_20_N_2_O_4_. MS *m*/*z* 341.13 (M + H^+^). ^1^H NMR (300 MHz, CDCl_3_) δ 7.79–7.87 (m, 2H), 7.66–7.75 (m, 2H), 7.21–7.27 (m, 2H), 6.80–6.88 (m, 2H), 4.01 (ddd, *J* = 7.05, 3.46, 1.54 Hz, 1H), 3.66–3.85 (m, 7H), 3.09 (br.s., 2H), 2.79 (dd, *J* = 12.57, 3.85 Hz, 1H), 2.64 (dd, *J* = 12.31, 7.44 Hz, 1H) ^13^C NMR (75 MHz, CDCl_3_) δ 168.63, 158.85, 134.02, 131.96, 131.05, 129.54, 123.35, 113.89, 67.75, 55.24, 52.95, 51.58, 41.88.

*2-(3-((2,4-Dichlorobenzyl)amino)-2-hydroxypropyl)isoindoline-1,3-dione* (**8**). Following the procedure A, reaction of (2,4-dichlorophenyl)methanamine (0.083 mL, 0.615 mmoL) with 2-(oxiran-2-ylmethyl)isoindoline-1,3-dione (**20**) (0.125 g, 0.615 mmol) and a catalytic amount of pyridine in 4.0 mL *n*-propanol was performed. Yield: 72 mg (31.4%), TLC (DCM/MeOH, 9.5/0.5, *v*/*v*) R_f_ = 0.25. MW 379.24. Formula C_18_H_16_Cl_2_N_2_O_3_. MS *m*/*z* 379.01, 380.00 (M + H^+^). ^1^H NMR (300 MHz, CDCl_3_) δ 7.79–7.88 (m, 2H), 7.68–7.76 (m, 2H), 7.35 (d, *J* = 2.05 Hz, 1H), 7.30 (d, *J* = 8.21 Hz, 1H), 7.19 (dd, *J* = 8.46, 2.31 Hz, 1H), 4.00 (tdd, *J* = 6.89, 6.89, 5.19, 4.10 Hz, 1H), 3.84–3.88 (m, 2H), 3.75–3.82 (m, 2H), 2.76 (dd, *J* = 12.57, 3.85 Hz, 1H), 2.63 (dd, *J* = 12.31, 6.92 Hz, 1H) 2.52 (br.s., 2H) ^13^C NMR (75 MHz, CDCl_3_) δ 168.69, 135.86, 134.40, 134.09, 133.43, 131.91, 130.80, 129.33, 127.10, 123.40, 68.24, 51.83, 50.57, 41.91.

*2-(4-(Benzylamino)-3-hydroxybutyl)isoindoline-1,3-dione* (**9**). Following the procedure A, reaction of phenylmethanamine (0.071 mL, 0.650 mmoL) with 2-(2-(oxiran-2-yl)ethyl)isoindoline-1,3-dione (**21**) (0.140 g, 0.650 mmoL) and a catalytic amount of pyridine in 4 mL *n*-propanol was performed. Yield: 55.2 mg (26.2%), TLC (DCM/MeOH, 9.5/0.5, *v*/*v*) R_f_ = 0.18. MW 324.38. Formula C_19_H_20_N_2_O_3_. MS *m*/*z* 325.11(M + H^+^). ^1^H NMR (300 MHz, CDCl_3_) δ 7.79–7.87 (m, 2H), 7.68–7.76 (m, 2H), 7.19–7.36 (m, 5H), 3.83–3.90 (m, 2H), 3.81 (s, 2H), 3.62–3.74 (m, 1H), 2.54–2.82 (m, 4H), 1.66–1.83 (m, 2H). ^13^C NMR (75 MHz, DMSO-*d*_6_) δ 168.35, 141.02, 134.73, 132.17, 128.52, 128.37, 127.00, 123.36, 67.89, 55.33, 53.36, 35.49, 33.95.

*2-(3-Hydroxy-4-((2-methoxybenzyl)amino)butyl)isoindoline-1,3-dione* (**10**). Following the procedure A, reaction of (2-methoxyphenyl)methanamine (0.072 mL, 0.550 mmoL) with 2-(2-(oxiran-2-yl)ethyl)isoindoline-1,3-dione (**21**) (0.120 g, 0.550 mmoL) and a catalytic amount of pyridine in 4 mL *n*-propanol was performed. Yield: 54 mg (27.7%), TLC (DCM/MeOH, 9.5/0.5, *v*/*v*) R_f_ = 0.21. MW 354.41. Formula C_20_H_22_N_2_O_4_. MS *m*/*z* 355.15 (M + H^+^). ^1^H NMR 7.77–7.87 (m, 2H), 7.65–7.75 (m, 2H), 7.27–7.38 (m, 2H), 6.85–6.99 (m, 2H), 4.16 (d, *J* = 13.48 Hz, 1H), 3.99 (d, *J* = 13.48 Hz, 1H), 3.86–3.92 (m, 3H), 3.69–3.86 (m, 4H), 2.90 (dd, *J* = 12.31, 2.93 Hz, 1H), 2.66 (dd, *J* = 12.31, 9.96 Hz, 1H), 1.50–1.86 (m, 3H). ^13^C NMR (75 MHz, CDCl_3_) δ 168.36, 157.46, 134.73, 132.17, 129.35, 128.35, 128.23, 123.36, 120.48, 110.85, 67.78, 55.63, 49.04, 47.97, 35.46, 33.95.

*2-(3-Hydroxy-4-((4-methoxybenzyl)amino)butyl)isoindoline-1,3-dione* (**11**). Following the procedure A, reaction of (4-methoxyphenyl)methanamine (0.096 mL, 0.738 mmoL) with 2-(2-(oxiran-2-yl)ethyl)isoindoline-1,3-dione (**21**) (130 mg, 0.600 mmoL) and a catalytic amount of pyridine in 3 mL *n*-propanol was performed. Yield: 55 mg (25.9%), TLC (DCM/MeOH, 9.5/0.5, *v*/*v*) R_f_ = 0.24. MW 354.41. Formula C_20_H_22_N_2_O_4_. MS *m*/*z* 355.08 (M + H^+^). ^1^H NMR (300 MHz, CDCl_3_) δ 7.77–7.87 (m, 2H), 7.66–7.74 (m, 2H), 7.24 (d, *J* = 8.46 Hz, 2H), 6.84 (d, *J* = 8.46 Hz, 2H), 3.84 (t, *J* = 6.67 Hz, 2H), 3.66–3.79 (m, 6H), 3.35 (br.s., 2H), 2.70 (dd, *J* = 11.80, 3.08 Hz, 1H), 2.58 (dd, *J* = 12.05, 8.72 Hz, 1H), 1.64–1.83 (m, 2H). ^13^C NMR (75 MHz, CDCl_3_) δ 168.67, 158.93, 133.98, 132.05, 130.49, 129.67, 123.27, 113.90, 66.85, 55.23, 53.94, 52.76, 34.63, 33.78.

*2-(5-(Benzylamino)-4-hydroxypentyl)isoindoline-1,3-dione* (**12**). Following the procedure A, reaction of phenylmethanamine (0.071 mL, 0.650 mmoL) with 2-(3-(oxiran-2-yl)propyl)isoindoline-1,3-dione (**22**) (0.150 g, 0.650 mmoL) and a catalytic amount of pyridine in 4 mL *n*-propanol was performed. Yield: 52 mg (23.7%), TLC (DCM/MeOH, 9.5/0.5, *v*/*v*) R_f_ = 0.21. MW 338.41. Formula C_20_H_22_N_2_O_3_. MS *m*/*z* 339.40 (M + H^+^). ^1^H NMR (300 MHz, CDCl_3_) δ 7.78–7.86 (m, 2H), 7.66–7.74 (m, 2H), 7.27–7.41 (m, 5H), 3.84–3.97 (m, 2H), 3.74–3.84 (m, 1H), 3.66–3.73 (m, 2H), 3.30 (br.s., 1H), 2.77 (dd, *J* = 12.31, 2.93 Hz, 1H), 2.55 (dd, *J* = 12.02, 9.67 Hz, 1H), 1.79–1.94 (m, 1H), 1.63–1.79 (m, 1H), 1.38–1.52 (m, 2H), 1.35 (d, *J* = 11.14 Hz, 1H). ^13^C NMR (75 MHz, CDCl_3_) δ 168.43, 137.05, 133.89, 132.08, 128.69, 127.82, 127.51, 123.18, 68.15, 53.86, 52.92, 37.76, 31.93, 24.81.

*2-(4-Hydroxy-5-((2-methoxybenzyl)amino)pentyl)isoindoline-1,3-dione* (**13**). Following the procedure A, reaction of (2-methoxyphenyl)methanamine (0.085 mL, 0.650 mmol) with 2-(3-(oxiran-2-yl)propyl)isoindoline-1,3-dione (**22**) (0.150 g, 0.650 mmol) and a catalytic amount of pyridine in 4 mL *n*-propanol was performed. Yield: 52 mg (21.8%), TLC (DCM/MeOH, 9.5/0.5, *v*/*v*) R_f_ = 0.18. MW 368.43. Formula C_21_H_24_N_2_O_3_. MS *m*/*z* 369.17 (M + H^+^). ^1^H NMR (300 MHz, CDCl_3_) δ 7.76–7.87 (m, 2H), 7.65–7.73 (m, 2H), 7.29–7.39 (m, 2H), 6.86–6.98 (m, 2H), 4.22 (d, *J* = 12.89 Hz, 1H), 4.01 (d, *J* = 12.89 Hz, 1H), 3.91 (s, 3H), 3.62–3.72 (m, 2H), 2.89 (dd, *J* = 12.31, 1.76 Hz, 1H), 2.62 (dd, *J* = 12.02, 10.26 Hz, 1H), 1.83 (dt, *J* = 9.23, 6.52 Hz, 1H), 1.58–1.75 (m, 1H), 1.31–1.44 (m, 3H), 1.23–1.30 (m, 2H). ^13^C NMR (75 MHz, CDCl_3_) δ 168.39, 157.81, 133.88, 132.05, 131.65, 131.10, 123.17, 120.88, 119.03, 110.64, 66.10, 55.59, 52.75, 47.77, 37.63, 31.73, 24.73.

*2-(6-(Benzylamino)-5-hydroxyhexyl)isoindoline-1,3-dione* (**14**). Following the procedure A, reaction of phenylmethanamine (0.062 mL, 0.570 mmoL) with 2-(4-(oxiran-2-yl)butyl)isoindoline-1,3-dione (**23**) (0.140 g, 0.570 mmoL) and a catalytic amount of pyridine in 4 mL *n*-propanol was performed. Yield: 69 mg (34.1%), TLC (DCM/MeOH, 9.5/0.5, *v*/*v*) R_f_ = 0.19. MW 352.43. Formula C_21_H_24_N_2_O_3_. MS *m*/*z* 353.22 (M + H^+^). ^1^H NMR (300 MHz, CDCl_3_) δ 7.77–7.86 (m, 2H), 7.65–7.73 (m, 2H), 7.27–7.44 (m, 5H), 3.88–4.01 (m, 2H), 3.70–3.84 (m, 2H), 3.66 (t, *J* = 7.03 Hz, 2H), 2.79 (dd, *J* = 12.31, 2.93 Hz, 1H), 2.58 (dd, *J* = 12.02, 9.67 Hz, 1H), 1.57–1.77 (m, 2H), 1.39–1.56 (m, 3H), 1.22–1.39 (m, 2H) ^13^C NMR (75 MHz, CDCl_3_) δ 168.42, 135.48, 133.86, 132.11, 129.09, 128.81, 128.23, 123.17, 68.03, 53.44, 52.54, 37.70, 34.31, 28.47, 22.68.

*2-(5-Hydroxy-6-((2-methoxybenzyl)amino)hexyl)isoindoline-1,3-dione* (**15**). Following the procedure A, reaction of (2-methoxyphenyl)methanamine (0.057 mL, 0.440 mmoL) with 2-(4-(oxiran-2-yl)butyl)isoindoline-1,3-dione (**23**) (0.109 g, 0.440 mmoL) and a catalytic amount of pyridine in 3 mL *n*-propanol was performed. Yield: 49.8 mg (29.4%), TLC (DCM/MeOH, 9.5/0.5, *v*/*v*) R_f_ = 0.16. MW 382.46. Formula C_22_H_26_N_2_O_3_. MS *m*/*z* 383.20 (M + H^+^). ^1^H NMR (300 MHz, CDCl_3_) δ 7.76–7.85 (m, 2H), 7.65–7.73 (m, 2H), 7.29–7.39 (m, 2H), 6.87–6.99 (m, 2H), 3.96–4.32 (m, 3H), 3.90 (s, 3H), 3.56–3.68 (m, 2H), 2.88 (dd, *J* = 12.31, 2.34 Hz, 1H), 2.62 (dd, *J* = 12.31, 9.96 Hz, 1H), 1.56–1.76 (m, 2H), 1.22–1.56 (m, 6H). ^13^C NMR (75 MHz, CDCl_3_) δ 168.36, 157.84, 133.85, 132.08, 131.74, 131.12, 123.15, 120.88, 118.88, 110.61, 66.30, 55.56, 52.52, 47.38, 37.69, 34.10, 28.41, 22.58.

### 3.2. Biological Activity

#### 3.2.1. In Vitro Inhibition of AChE and BuChE

Inhibitory activities of the synthesized compounds against the cholinesterases were measured using the spectrophotometric method described by Ellman et al. [23], as modified for 96-well microplates. AChE from *Electrophorus electricus* (*ee*AChE) and BuChE from equine serum (*eq*BuChE) were used. 5,5′-Dithiobis-(2-nitrobenzoic acid) (DTNB), acetylthiocholine iodide (ATC), butyrylthiocholine iodide (BTC), and both cholinesterases were purchased from Sigma–Aldrich. The enzymes were prepared by dissolving 500 U of each in water to give stock solutions of 5 U/mL. *ee*AChE and *eq*BuChE were further diluted before use to a final concentration of 0.384 U/mL. In the first step of Ellman’s method, 25 μL of the test compound (or water; i.e., blank samples) was incubated in 0.1 M phosphate buffer (200 μL, pH = 8.0) with DTNB (20 μL; 0.0025 M) and the enzyme (20 μL; *ee*AChE or *eq*BuChE) at a temperature of 25 °C. Then, after an incubation period of 5 min, 20 μL of ATC (0.00375 M) or BTC (0.00375 M) solutions (depending on the enzyme used) were added. After a further 5 min, the changes in absorbance were measured at 412 nm using a microplate reader (EnSpire Multimode; PerkinElmer, Hong Kong, China). All compounds were tested at a screening concentration of 10 μM. Percent of inhibition was calculated from 100 − (S/B) × 100, where S and B were the respective enzyme activities with and without the test sample, respectively. For compounds with better than 50% inhibitory activity at 10 μM, the IC_50_ values were determined. To determine the IC_50_ value, six different concentrations of each compound were used to obtain enzyme activities between 5% and 95%. All reactions were performed in triplicate. The IC_50_ values were calculated using nonlinear regression (GraphPad Prism 5 (GraphPad Software, San Diego, CA, USA, 5.00)) by plotting the residual enzyme activities against the applied inhibitor concentration.

#### 3.2.2. In Vitro BACE-1 Inhibitory Activity

All the compounds were tested as *h*BACE-1 inhibitors using a PanVera’s BACE-1 fluorescence resonance energy transfer (FRET) [24,25] Assay Kit, which includes purified baclovirus-expressed BACE-1 and specific peptide substrate (Rh-EVNLDAEFK-quencher), based on the “Swedish” mutant of APP. The assay was purchased from Life Technologies Polska Sp. z o.o. The analyses were carried out according to the supplied protocol with small modifications using 384-well black microplates and a microplate reader (EnSpire Multimode; PerkinElmer).The wavelength was optimized for 553 nm excitation and 576 nm emission. Stock solutions of all test compounds were prepared in DMSO and further diluted with assay buffer (50 mM sodium acetate; pH 4.5). In the first step, 10 μL of BACE-1 substrate was mixed with 10 μL of test compound (or assay buffer; i.e., blank sample), then 10 μL of enzyme (1 U/mL) was added to start the reaction. After 60 min of incubation at 25 °C, 10 μL of stop solution (2.5 M sodium acetate) was applied to stop the reaction. The fluorescence signal was read at 576 nm. Percent of inhibition was calculated from [1 − (S60 − S0)/(C60 − C0)] × 100, where S0 and S60 are the fluorescence intensities of the test sample (enzyme, substrate, test compound) at the beginning of the reaction and after 60 min, respectively, while C0 and C60 are the analogical fluorescence intensities of the blank sample (enzyme, substrate, buffer). All of the compounds were tested at a screening concentration of 50 μM. Each compound was analyzed in triplicate. BACE-1 Inhibitor **IV** (Calbiochem, Merck, Nottingham, UK) was used as the reference compound.

#### 3.2.3. Inhibition of Aβ-Aggregation

In order to investigate the inhibition of β-amyloid peptide aggregation by the compounds, a thioflavin T-based fluorometric assay was performed [27]. Recombinant human HFIP-pretreated Aβ_1–42_ peptide (Lot 2691412 and 2718332, Merck Millipore, Darmstadt, Germany) was dissolved in DMSO to give a 75 μM stock solution. The stock solution was further diluted in HEPES buffered solution (150 mM HEPES, pH 7.4, 150 mM NaCl) to 7.5 μM. Aβ_1–42_ solution was then added to the test compounds in black-walled 96-well plate and diluted with ThT solution (final concentration of 10 μM). The final mixture contained 1.5 μM Aβ_1–42_, 10 μM of test compound, and 3% DMSO. ThT fluorescence was measured every 300 s (excitation wavelength of 440 nm, emission wavelength of 490 nm), with the medium continuously shaking between measurements using a 96-well microplate reader (Synergy™ H4, BioTek Instruments, Inc., Weinusky, VT, USA). The ThT emission of the Aβ_1–42_ began to rise after approximately 4 h, reached a plateau after 20 h, and remained almost unchanged for an additional 28 h of incubation. The fluorescence intensities at the plateau in the absence and presence of the test compounds were averaged, and the average fluorescence of the corresponding wells at t = 0 h was subtracted. The Aβ_1–42_ aggregation inhibitory potency is expressed as the percentage inhibition (% inh = (1 − Fi / F0) × 100%), where Fi is the increase in fluorescence of Aβ_1–42_ treated with the test compound and F0 is the increase in fluorescence of Aβ_1–42_ alone. The percentage of inhibition is expressed as mean ± standard deviation of at least two independent experiments; *p* < 0.05, statistically different compared to control experiments (Aβ_1–42_ + DMSO); one-way analysis of variance (ANOVA), followed by *post hoc* Bonferroni *t*-test (SigmaPlot v 12.0).

### 3.3. Molecular Modeling

All compounds prepared by the LigPrep program were docked with GOLD 5.3 (CCDC) to AChE (PDB code 1EVE), BuChE (PDB code 1P0I) complex, and to BACE-1 (PDB code 4D8C) [37]. Each protein was prepared using Hermes 1.7 (CCDC) [38]. All histidine residues were protonated at Nε and the hydrogen atoms were added. The binding sites were defined as: all amino acid residues within a radius of 10 Å from the donepezil (E20) present in the AChE, all amino acid residues within a radius of 20 Å from the glycerol molecule (GOL) present in the active center of BuChE, and all amino acid residues within a radius of 10 Å from the ligand (BXD) in the BACE-1 complex. Standard settings of the genetic algorithm were applied in each docking procedure. We received 10 conformations for each docked ligand sorted by the ChemScore (for AChE and BuChE) and the GoldScore (for BACE-1) function value. Results of docking were visualized with PyMOL 0.99rc6 (DeLano Scientific LLC) [39].

## 4. Conclusions

Modification of the lead structure is a simple way to improve affinity for a selected (desired) biological target. In the case of multi-target-directed ligands, this strategy is more complicated because it requires optimization towards more than one target. In this study, new potential MTDLs have been designed and synthesized based on previously obtained AChE inhibitors. The structure of lead **III** (2-(5-(4-flurobenzylamino)pentyl)isoindoline-1,3-dione) was modified with expected activity against disease-modifying and symptomatic targets. In order to introduce additional inhibitory activity against BACE-1, we enriched the base compound by adding a hydroxyl group at position 2 of the alkyl chain, which is a common moiety occurring in BACE-1 inhibitors. Results indicate that the presented modification leads to an overall decrease in anticholinesterase activity; however, an inhibitory effect against BACE-1 appears. In terms of cholinesterase inhibition, the most interesting compounds are **5** (selective inhibitor of *eq*BuChE) and **15** (dual action against both cholinesterases). However, considering the multifunctional profile, the most valuable compound seems to be **12** (2-(5-(benzylamino)-4-hydroxypentyl)isoindoline-1,3-dione) with a balanced activity profile involving inhibition of *ee*AChE (IC_50_ = 3.33 μM), *h*BACE-1(43.7% at 50 μM), and Aβ-aggregation (24.9% at 10 μM). Most existing multifunctional, anti-Alzheimer’s agents are cholinesterase inhibitors with some additional properties, such as inhibition of Aμ aggregation, antioxidative action, a neuroprotective effect, or metal chelating activity. Relatively few inhibitors of both cholinesterase and BACE-1 have been identified thus far. Therefore, the presented results represent a promising start towards the identification and synthesis of new active multifunctional agents.

## Figures and Tables

**Table 1:** Inhibitory activity on *ee*AChE, *eq*BuChE, *h*BACE-1, and Aβ for the presented compounds.

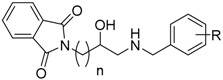						
Cmp.	R	*n*	*ee*AChE ^a^	*eq*BuChE ^b^	*h*BACE-1 ^c.^	Aβ-Aggr. ^d^
			IC_50_ (µM) ^e^/% Inh. ^f^	IC_50_ (µM) ^e^/% Inh. ^f^	% Inh. ^f^	% Inh. ^f^
**1**	H	1	11.5% ± 5.0%	14.1% ± 3.3%	37.3% ± 7.5%	<10%
**2**	2-F	1	28.1% ± 2.0%	19.5% ± 2.0%	39.6% ± 6.5%	21.1% ± 6.6%
**3**	2-CF3	1	11.8% ± 5.0%	<10%	41.8% ± 4.5%	<10%
**4**	3-Cl	1	<10%	24.1% ± 4.7%	34.1% ± 7.7%	<10%
**5**	2-OMe	1	<10%	7.86 ± 0.29	39.2% ± 9.2%	19.9% ± 1.5%
**6**	4-Me	1	<10%	11.6% ± 3.3%	45.0% ± 2.0%	<10%
**7**	4-OMe	1	<10%	19.1% ± 1.5%	35.1% ± 10.0%	<10%
**8**	2,4-diCl	1	<10%	21.7% ± 1.0%	37.2% ± 1.3%	<10%
**9**	H	2	<10%	<10%	<10%	<10%
**10**	2-OMe	2	32.7% ± 4.7%	17.8% ± 2.0%	24.4% ± 5.9%	<10%
**11**	4-OMe	2	<10%	<10%	34.8% ± 10.6%	<10%
**12**	H	3	3.32 ± 0.15	14.1% ± 5.8%	43.7% ± 6.9%	24.9% ± 6.6%
**13**	2-OMe	3	11.07 ± 0.49	26.2% ± 9.7%	37.1% ± 3.9%	<10%
**14**	H	4	2.13 ± 0.07	36.0% ± 7.2%	42.1% ± 10.7%	<10%
**15**	2-OMe	4	1.95 ± 0.06	36.0% ± 1.1%	15.7% ± 1.3%	<10%
Reference compounds						
Donepezil			0.011 ± 0.0002	1.83 ± 0.04	n.d. ^g^	13.8% ± 6.8
Tacrine			0.023 ± 0.0004	0.015 ± 0.0001	n.d. ^g^	n.d. ^g^
Inhibitor IV ^h^			n.d. ^g^	n.d. ^g^	0.046 ± 0.001 ^i^	n.d. ^g^
Resveratrol			n.d. ^g^	n.d. ^g^	n.d. ^g^	78.5% ± 5.2

^a^ IC_50_ inhibitory concentration of AChE from electric eel or inhibition percentage at 10 μM inhibitor concentration. ^b^ IC_50_ inhibitory concentration of BuChE from horse serum or inhibition percentage at 10 μM inhibitor concentration. ^c^ inhibition percentage of *h*BACE-1 at 50 μM inhibitor concentration. ^d^ inhibition percentage of Aβ-aggregation at 10 μM inhibitor concentration. ^e^ Values are expressed as means ± standard error of the mean (SEM) for at least three experiments. ^f^ Values are expressed as means ± standard deviation (SD) for at least two independent experiments. ^g^ Not determined. ^h^ Calbiochem, Merck; Nottingham, UK. ^i^ IC_50_ value.
